# Supplementary material for: Mono-Dispersed Microspheres Locally Assembled on Porous Substrates Formed through a Microemulsion Approach
Source: Polymers (Basel). 2020 Apr 21;12(4):964. doi: 10.3390/polym12040964 (PMC7240494; doi:10.3390/polym12040964)
Supplement: Supplementary file 1 [file polymers-12-00964-s001.pdf]

Supporting Information

Mono-Dispersed Microspheres Locally Assembled on Porous Substrates Formed through Microemulsion Approach

Jianfeng Zhang, Shuxin Gong, Jiahang Zhu, Jiejing Zhang, Jing Liang*

College of life science, Key Laboratory of Straw Biology and Utilization, the Ministry of Education, Jilin Agricultural University, Changchun 130118, China; zhangjianfeng06@tsinghua.org.cn (J.Z.); gsx1127117149@163.com (S.G.); zhujiahangzhujia@163.com (J.Z.); zjjx124@163.com (J.Z.)

* Correspondence: liangjing@jlau.edu.cn

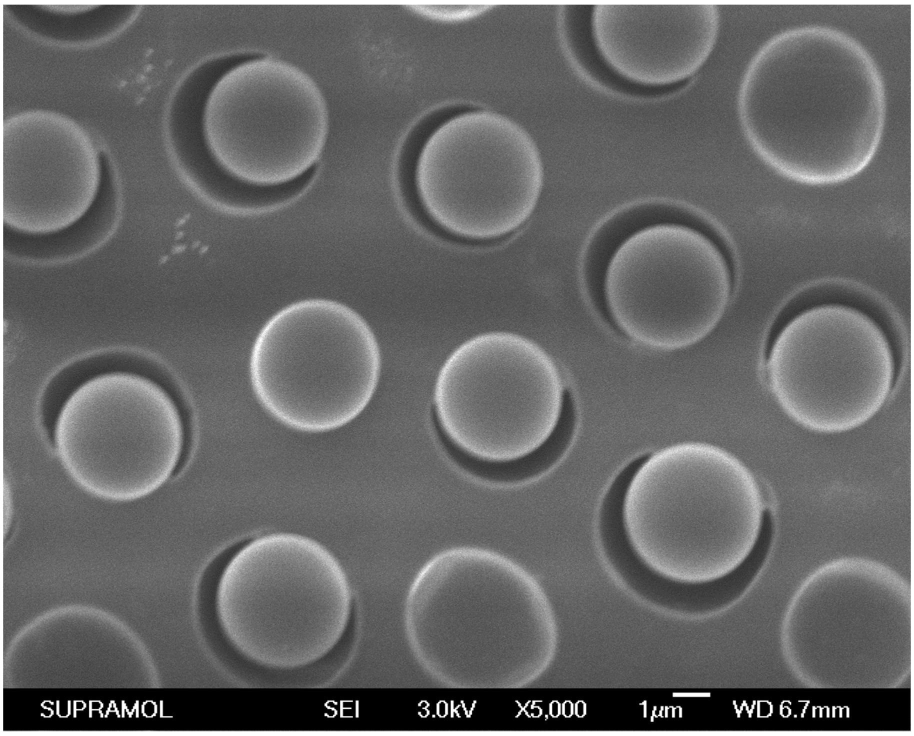

Figure S1. SEM images of porous films filled of PS microspheres with the content 0.1% w/v after washing with water.
